# Stochastic demethylation and redundant epigenetic suppressive mechanisms generate highly heterogeneous responses to pharmacological DNA methyltransferase inhibition

**DOI:** 10.1186/s13046-025-03294-x

**Published:** 2025-01-23

**Authors:** Mie K. Jakobsen, Sofie Traynor, Aaraby Y. Nielsen, Christina Dahl, Mette Staehr, Simon T. Jakobsen, Maria S. Madsen, Rasmus Siersbaek, Mikkel G. Terp, Josefine B. Jensen, Christina B. Pedersen, Anup Shrestha, Jonathan R. Brewer, Pascal H. G. Duijf, Odd L. Gammelgaard, Henrik J. Ditzel, Alexei F. Kirkin, Per Guldberg, Morten F. Gjerstorff

**Affiliations:** 1 Department of Cancer Research, Institute of Molecular Medicine, University of Southern Denmark, Odense, Denmark; 2 Danish Cancer Institute, Copenhagen, Denmark; 3 Department of Biochemistry and Molecular Biology, University of Southern Denmark, Odense, Denmark; 4 Centre for Cancer Biology, Clinical and Health Sciences, University of South Australia & SA Pathology, Adelaide, SA Australia; 5 Institute of Clinical Medicine, Faculty of Medicine, University of Oslo, Oslo, Norway; 6 Department of Medical Genetics, Oslo University Hospital, Oslo, Norway; 7 Department of Oncology, Odense University Hospital, Odense, Denmark; 8 Academy of Geriatric Cancer Research (AgeCare), Odense University Hospital, Odense, Denmark; 9 Cytovac A/S, Hørsholm, 2970 Denmark

**Keywords:** DNA methylation, DNA methyltransferase inhibitor, Cancer/testis antigen.

## Abstract

**Background:**

Despite promising preclinical studies, the application of DNA methyltransferase inhibitors in treating patients with solid cancers has thus far produced only modest outcomes. The presence of intratumoral heterogeneity in response to DNA methyltransferase inhibitors could significantly influence clinical efficacy, yet our understanding of the single-cell response to these drugs in solid tumors remains very limited.

**Methods:**

In this study, we used cancer/testis antigen genes as a model for methylation-dependent gene expression to examine the activity of DNA methyltransferase inhibitors and their potential synergistic effect with histone deacetylase inhibitors at the single-cancer cell level. The analysis was performed on breast cancer patient-derived xenograft tumors and cell lines, employing a comprehensive set of techniques, including targeted single-cell mRNA sequencing. Mechanistic insights were further gained through DNA methylation profiling and chromatin structure analysis.

**Results:**

We show that breast cancer tumors and cell cultures exhibit a highly heterogenous response to DNA methyltransferase inhibitors, persisting even under high drug concentrations and efficient DNA methyltransferase depletion. The observed variability in response to DNA methyltransferase inhibitors was independent of cancer-associated aberrations and clonal genetic diversity. Instead, these variations were attributed to stochastic demethylation of regulatory CpG sites and the DNA methylation-independent suppressive function of histone deacetylases.

**Conclusions:**

Our findings point to intratumoral heterogeneity as a limiting factor in the use of DNA methyltransferase inhibitors as single agents in treatment of solid cancers and highlight histone deacetylase inhibitors as essential partners to DNA methyltransferase inhibitors in the clinic.

**Supplementary Information:**

The online version contains supplementary material available at 10.1186/s13046-025-03294-x.

## Background

Alterations in DNA methylation patterns represent an ubiquitous characteristic of cancer [1, 2] and reflect the molecular evolution of tumors [3–6]. These patterns involve hypermethylation events that facilitate cancer development, progression, and drug resistance by silencing specific genes [2]. Importantly, drugs like decitabine and guadecitabine, which inhibit the activity of DNA methyltransferases (DNMTs), can reverse hypermethylation in cancer cells. These agents substitute cytosine in DNA synthesis, thereby directly impeding the perpetuation of DNA methylation marks during replication. Additionally, they covalently entrap and facilitate the degradation of the three catalytically active DNMT enzymes: DNMT1, DNMT3A, and DNMT3B [2, 7].

A substantial body of preclinical research suggests that DNMT inhibitors (DNMTis) hold significant promise as treatments for solid tumors. By reversing cancer-associated hypermethylation, DNMTis can re-induce the expression of tumor suppressor genes [8–10] and genes critical for antigen presentation [11–13]. Additionally, DNMTi treatment can activate genes that are typically silenced by DNA methylation in somatic cells, such as those encoding human endogenous retroviruses (ERVs) [14, 15] and cancer/testis antigens (CTAs) [16]. The transcription of double-stranded RNAs from otherwise latent human ERVs promotes a state of viral mimicry, activating intracellular viral response pathways and production of pro-inflammatory cytokines [14, 15, 17, 18]. CTAs comprise a large group of structurally and functionally diverse proteins, which are exclusively expressed in testicular germ cells under non-pathogenic conditions. Central tolerance is sub-optimal rendering many CTAs immunogenic when expressed in cancers [19, 20]. In line with these molecular findings, DNMTis have demonstrated impressive effects on preclinical models of solid cancer, including management of tumor growth and metastasis, as well as the enhancement anti-tumor immunity [21–23]. Furthermore, DNMTis can sensitize cancer cells to chemotherapeutics or targeted drugs, thereby limiting drug resistance [24–28], and amplify the effects of immune checkpoint inhibition [22, 29–31].

Despite encouraging results in preclinical studies, the use of DNMTis for treating patients with solid cancers has yielded only modest response rates [32], and the mechanism of therapeutic resistance remains largely uncharacterized. Intratumoral heterogeneity has a profound effect on clinical drug responses and may also limit the clinical potential of DNMTis. However, our understanding of how individual cancer cells react to DNMTis remains inadequate. In this study, we employed the activation of CTA gene expression as a measure to assess responses to DNMTi therapy at the single-cell level, allowing us to thoroughly characterize, and establish strategies for limiting, inter-cellular heterogeneity in the response.

## Methods

### Cell culture

MBA-MB-231 and MCF-7 cells were cultured in Dulbecco’s Modified Eagle Medium (DMEM, Sigma-Aldrich), SK-BR-3 was cultured in McCoy’s 5 A Medium (Sigma-Aldrich) and ZR-75-1 and T-47D cells were cultured in Rosswell Park Memorial Institute (RPMI) 1640 Medium (Sigma-Aldrich). Cell lines were obtained from the American Type Culture Collection. Growth media were supplemented with 10% fetal bovine serum (FBS, Sigma-Aldrich) and 1% penicillin-streptomycin and cells were incubated at 37 °C and 5% CO_2_. Mycoplasma tests were performed regularly (MycoAlert, Mycoplasma detection kit, Lonza) and cell identities according to ATCC were verified using DNA fingerprinting by short tandem repeat (STR) analysis (Cell IDTM system). Cells were treated with the DNMTi guadecitabine (SGI-110; Selleckchem) and/or with the HDACi entinostat (Selleckchem)/valproic acid (Sigma-Aldrich) using the treatment schedules and concentrations indicated in figure legends.

### TNBC PDX xenograft models

Tissue biopsies were collected from TNBC patients undergoing routine treatment at Odense University Hospital. All patient samples were collected in compliance with informed consent policy and coded to maintain patient confidentiality. Fresh tissue or frozen stocks of PDX tumors were implanted with Matrigel (Sigma-Aldrich) into the mammary fat pad of 7–8-week-old female NOG CIEA mice (NOD.Cg-*Prkdcscid Il2rgtm1Sug*/JicTac, Taconic) housed under pathogen-free conditions with *ad libitum* food and water [33]. PDX models PDX-4582, -5160, and − 5474 were established from primary tumors of untreated patients, while PDX-9228 was established from a metastasis. PDX tumor samples were stored in DMEM with 10% DMSO in liquid nitrogen and used in low passage (second or third generation). Please refer to our previous publications for a more thorough description of the PDX models [17, 33]. When tumors were palpable (approximately 2–3 mm), mice were treated with subcutaneous injections of guadecitabine using two different treatment regimens: (1) 2 mg/kg guadecitabine 5 days a week for two weeks (low-dose schedule), and (2) 24.4 mg/kg guadecitabine every 5 days for 20 days (high-dose schedule). These dosing schedules were based on data on the pharmacokinetic properties and effect on methylation and expression of *LINE-1* and CTA genes in various xenograft models [34–36]. Mice were euthanized by cervical dislocation 3 days after the last treatment and tumors were processed for RNA-sequencing and/or immunohistochemical staining. The experiments were approved by the Danish Animal Experiments Inspectorate.

### Bioinformatic analysis of CTA expression in clinical TNBC tumors

Gene expression levels for triple-negative breast cancer (TNBC) samples were obtained from the RNAseq (polyA + IlluminaHiSeq) platform of The Cancer Genome Atlas (TCGA) breast invasive carcinoma (BRCA) gene expression dataset. The TCGA consortium experimentally measured gene expression profiles using the Illumina HiSeq 2000 RNA-sequencing platform. Level 3 data were downloaded from the TCGA Data Coordination Center by querying the Cancer Genomic Data Server (CGDS) using the *R*/*CGDS* package. Based on matched clinical data, TNBC samples were selected from the BRCA pan-cancer atlas samples. Normalized gene-level transcription estimates were log-transformed using the following equation: $\:g={log}_{2}\left(nc+1\right)$. Herein, $\:g$ represents the gene-level transcription estimate and $\:nc$ denotes the RNA-Seq by Expectation Maximization (RSEM)-normalized read counts.

### Quantitative real time-PCR

RNA was purified using the RiboZol (VWR) kit and used for cDNA synthesis with the RevertAid Premium Reverse Transcriptase kit from Fermentas. Quantitative real-time (RT) PCR was performed using SYBR green-based expression analysis (Applied Biosystems). QuantiTect primers used for RT-PCR analysis were: *MAGEA3*; QT00064799, *MAGEC1*; QT00206955, *CTAG1B*; QT00088956 (Qiagen).

### RNA sequencing

RNA was purified from cells using RiboZol (VWR) and tissues using TRI Reagent (Sigma-Aldrich). For tumor tissues, 2.8 mm Zirconium oxide beads (Precellus) were added, and homogenization was carried out using a Precellus 24 homogenizer (3 × 15 s, 6500 rpm). Preparation of sequencing libraries was done using NEBNext Poly(A) mRNA Magnetic Isolation Module (New England Biolabs) and the NEBNext Ultra II DNA Library Prep Kit for Illumina (New England Biolabs) with unique dual indexes according to manufacturer’s instructions. Libraries were sequenced on an Illumina NovaSeq 6000 Sequencing system. Trimmed and filtered sequencing reads were aligned to the human genome (hg38) using “Spliced Transcripts Alignment to a Reference” (STAR [37]) and analyzed using the Qlucore analysis platform (Qlucore) and in R using the R/DESeq2 package [38].

### Immunohistochemistry

Tumors and cell line aggregates, made by mixing with plasma and thrombin, were fixed in 10% neutral buffered formalin and embedded in paraffin. Paraffin blocks were cut into 10 μm sections with a microtome, mounted on ChemMateTM Capillary Gap Slides (Dako), dried at 60 °C, deparaffinized, and hydrated. Endogenous peroxidase activity was blocked with 1.5% hydrogen peroxide in TBS buffer (pH 7.4) for 10 min. Antigen-retrieval was carried out by microwave boiling in T-EG buffer (Dako) for 15 min and sections were incubated with the primary antibodies (Supplementary Methods 1) at room temperature for 32 min before development with EnVision FLEX HRP (Agilent).

### Quantitative immunofluorescence

Cells were grown on coverslips, fixed in 4% formaldehyde and permeabilized in 0.2% Triton X-100 (Sigma-Aldrich). Subsequently, cells were blocked in 3% BSA in PBS before incubation with primary antibodies (Supplementary Methods 1) for 90 min at room temperature, washed, and incubated in the dark at RT with secondary anti-mouse and anti-rabbit antibodies (AlexaFlour 488, A11034 and AlexaFlour 568, A11031, ThermoFisher Scientific) for 60 min and DAPI for 5 min. Lastly, coverslips were dried and placed upside-down on microscope slides containing antifade (ProLong Gold antifade reagent, ThermoFisher Scientific). The images of stained MDA-MB-231 and MCF-cells were acquired with the Nikon Ti2 widefield microscope, equipped with CoolLED pE-300 white light source and Andor Zyla sCMOS camera. Images were acquired on random spots on the coverslips using random point generator function in the Nikon NIS element software. The acquired images were analyzed using the NIS -Elements AR software. General Analysis 3 (GA3) feature was used for segmentation and the calculation of the mean intensities for various channels. The segmented masks created from the nuclear channel (DAPI) was applied to other channels (Alexa Fluor 488 and Alexa Fluor 568) to calculate the mean intensities. Image acquisition and image analysis were performed at the Danish Molecular Biomedical Imaging Center (DaMBIC, University of Southern Denmark).

### Edu-labeling

MDA-MB-231 cells were treated with either 1 µM guadecitabine or vehicle for 48 h before being labeled with 10 µM EdU for 24 h using the Click-iT EdU Imaging Kit (ThermoFisher Scientific) according to manufacturer’s instructions. Subsequently, cells were fixed in 4% formaldehyde and permeabilized in 0.2% Triton X-100 (Sigma-Aldrich) before EdU detection according to manufacturer’s instructions and image capture using an IX-73 Olympus fluorescence microscope. Mean intensity of EdU in nuclei was performed using ImageJ software.

### PKH26 cell division assay

MDA-MB-231 cells were stained with PKH26 (PKH26 Red Fluorescent Cell Linker Kits for General Cell Membrane Labelling, MINI26, Sigma-Aldrich) for 5 min in the dark at room temperature and blocked with FBS, before three steps of washing in complete media, according to manufacturer’s instructions. Subsequently, PKH26-stained cells were treated with either 0.1 µM guadecitabine or vehicle for five days before being sorted on a Becton Dickinson FACSAria III Cell Sorter and Analyzer based on number of cell divisions, according to PKH26 dilution in the cell membrane. Lastly, RNA purification and sequencing were performed as described above.

### Production of single-cell MDA-MB-231 clones

Single MDA-MB-231 cells were sorted into wells of 96 well plates using a FACSAria cell sorter (BD Biosciences). Cells were expanded and analyzed at low passage.

### DNMTi treatment of CD4 + T cells

CD4 + T cells were prepared as previously described [39]. In brief, a mixture of autologous non-adherent lymphocytes and mature DCs (in a 10:1 ratio) was incubated in AIM-V medium containing 1% autologous serum. At day 1, 4 and 6 fresh medium containing IL-2 at a final concentration of 25 IU/ml was added. On day 7, cells were collected, counted, washed, and resuspended in media containing 150 IU/ml IL-2 and 10 µM guadecitabine and subsequently cultured for two days.

### Single-cell RNA-Sequencing and analysis

Based on RNA-seq data from breast cancer clinical tumors, PDX tumors and cell lines, we selected a panel of 83 CTA genes for targeted single-cell RNA-sequencing analysis (Supplementary methods 2). Primer libraries targeting these CTA genes were purchased from BD Biosciences. CD4 + T cells, MDA-MB-231 cells and MCF-7 cells were counted using the BD Rhapsody scanner (BD Biosciences) and individual samples were labeled with sample tags (BD Human Single-Cell Multiplexing Kit, 633781) before we performed single cell capture, cell lyses and reverse transcription with the BD Rhapsody Single-Cell Analysis System according to manufacturer’s instructions. Finally, mRNA libraries were prepared using the BD Rhapsody Targeted mRNA and AbSeq Amplification Kit (BD Biosciences) in combination with custom primer libraries for amplification of target CTA genes (BD Biosciences) and the BD Rhapsody Onco-BC targeted panel (BD Biosciences) for analysis of 389 breast cancer-related genes. In this context, the latter was used to identify CTA-negative subpopulations of cells. The libraries were quantified using a Qubit Fluorometer with the Qubit dsDNA HS Assay Kit (Thermo Fisher Scientific, Q32851) and size-distribution was analyzed using the Agilent DNA High Sensitivity Kit (Agilent Technologies, 5067 − 4626) on a TapeStation 4200 system. Libraries were sequenced in paired-end mode (2 × 75 bp) with 20% PhiX Spike-in on an Illumina Novaseq System. Basecalling and demultiplexing were performed by bcl2fastq 2.20. Quality filtering, mapping, putative cell calling and distribution based UMI error correction were performed using the BD Rhapsody Targeted Analysis Pipeline v1.11.1 on the SevenBridges platform. Expression matrices were further analyzed using the R/Seurat 4.3.0 package [40].

### Pyrosequencing

Genomic DNA (approximately 1 µg) was treated with bisulfite using the EZ DNA Methylation Kit (Zymo Research), according to the manufacturer’s protocol. The methylation status of *SSX1*, *PAGE5*, *DDX43*, and *MAGE2B* was assessed via pyrosequencing by bisulfite-converted DNA, using the PyroMark PCR Master Mix (Qiagen) with a final MgCl_2_ concentration of 1.5 mmol/L and primer concentrations of 200 nmol/L. Pyrosequencing analysis was performed on the PyroMark Q24 Instrument (Qiagen), and data was processed using the PyroMark software (Qiagen). Only samples passing the internal quality control assessment using standard settings were included in the analysis. Each experiment included positive controls for methylation (bisulfite-treated, in vitro methylated DNA; Universal Methylated DNA Standard, Zymo Research (IVM)), negative controls for methylation (bisulfite-treated, whole genome-amplified DNA (WGA)), and a no-template control. Primer sequences are listed in Supplementary Methods 1.

### Cloning and methylation analysis

Purification of genomic DNA from guadecitabine or vehicle treated MDA-MB-231 cells was performed using the Wizard SV Genomic DNA purification system (Promega) according to the manufacturer’s instructions. Bisulfite conversion of purified gDNA was performed using the EpiJET Bisulfite Conversion Kit (Thermo Scientific) according to the manufacturer’s instructions. Bisulfite-specific primers were designed using MethPrimer (https://www.urogene.org/methprimer/) [41]. To facilitate their binding to both methylated and unmethylated sequences, the primers were designed to avoid any CpGs. Primers are listed in supplementary methods. PCR was performed using 0.5 U Platinum Taq Polymerase High Fidelity (Thermo Fischer, Cat. 11304-011) and 50 ng converted DNA, according to the manufacturer’s recommendations. Amplicons were ligated into the pCR™4-TOPO™ vector using the TOPO™ TA Cloning™ Kit for Sequencing (Thermo Fischer) according to manufacturer’s instructions and ligation was introduced into One Shot^®^ TOP10 and DH5α™-T1R competent cells by heat shock. Transformed clones were grown on LB plates containing 50 µg/mL ampicillin. Individual colonies were subjected to Sanger sequencing (Eurofins) and subsequent analysis using SnapGene software to determine CpG methylation status.

### ATAC-seq

ATAC-seq was performed in two independent biological replicates. Cells were lysed in resuspension buffer (10mM Tris-HCL pH 7.4, 10mM NaCl, 3mM MgCl_2_ and 0.1% Tween-20) with the addition of 0.1% IgePal-CA630 and 0.01% Digitonin, followed by three washed in the resuspension buffer. Nuclei were pelleted and resuspended in 50uL tagmentation buffer (25 µl TD 2x reaction buffer, 0.5 µl 1% digitonin, 0.5 µl 10% Tween-20 and 1.25 µl TDE1 (Illumina, 20034197) and Nuclease Free water to 50 µl), and incubated at 37^o^C for 30 min. DNA was purified with Qiagen PCR Purification kit before PCR amplification for 10 cycles using Q5 High Fidelity 2x mater mix (New England BioLabs M0492) and the appropriate Illumina index primers. Samples were size selected using AMpure XP beads (Backman Coulter, A63881). Next Generation sequencing was performed on the NocaSeq6000 to obtain ~ 25 M 50 bp paired end reads per sample. ATAC-seq data were aligned to the human genome using STAR [37]. Polymerase chain reaction (PCR) duplicated reads were removed using samtools [42] and MACS2 [43] were used with default settings to call peaks. Only peaks called in both biological replicates were considered for downstream analysis. DiffBind [44] was used to call differential chromatin accessibility and Homer [45] was used to create average plots.

### Statistical analysis

Statistical testing was performed in GraphPad Prism v8 (GraphPad Software, Inc.) using either a two-tailed Student’s *t*-test or a one-way analysis of variance (ANOVA) as indicated in the figure legends.

## Results

### Intratumoral heterogeneity in DNMTi-mediated gene activation in breast cancers

DNMTis show promise as treatments for solid cancers, including breast cancer, where they increase the expression and presentation of CTAs and viral tumor antigens, activate interferon antiviral signaling, and enhance the response to endocrine therapy [11, 14, 15, 17, 46, 47]. To examine the clinical utility of DNMTis in the management of solid tumors, a comprehensive understanding of the intratumoral heterogeneity in response to these agents is essential. To address this knowledge gap, we investigated the response to DNMTi administration in breast cancer models, both in vitro and in vivo, employing the upregulation of CTA gene expression as a surrogate marker for assessing therapeutic efficacy at the single-cell resolution.

**Fig. 1:**
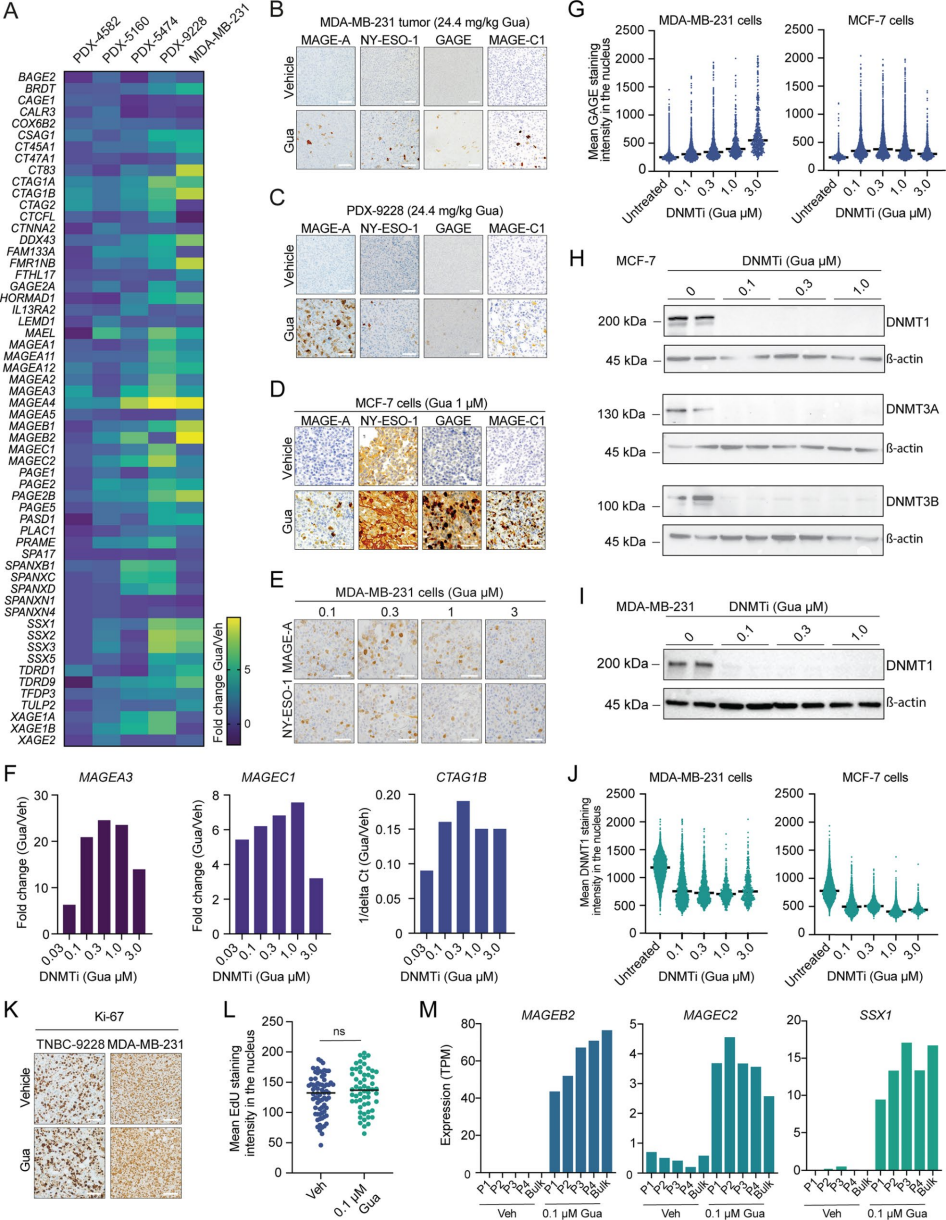
Heterogenous activation of CTA expression in breast cancer tumors and cell lines by DNMTi treatment. (**A**) RNA-sequencing analysis of CTA expression in breast cancer PDX tumors and MDA-MB-231 cells treated with guadecitabine (Gua)- or vehicle (Veh)-treated every five days for a total of four treatments. Four biological replicates were analyzed. (**B-C**) Immunohistochemical staining of selected CTA genes (brown) in MDA-MB-231 (**B**) and 9228 PDX breast cancer tumors (**C**) treated with guadecitabine or vehicle. Five biological replicates were analyzed. Representative images are shown. Counterstain: Hematoxylin (blue). Size bars = 100 µM. (**D-E**) Immunohistochemical staining of selected CTA genes (brown) in MCF-7 and MDA-MB-231 cells treated with indicated concentrations of guadecitabine or vehicle in vitro for 6 days. The analysis was performed in biological duplicates. Representative images are shown. Counterstain: Hematoxylin (blue). Size bars = 100 µM. **(F)** qPCR analysis of *MAGE-A3*,* MAGE-C1* and *CTAG1B* gene expression in MDA-MB-231 cells treated with the indicated concentrations of guadecitabine for 6 days. Histograms depict the average of two biological replicates relative to the expression in vehicle-treated MDA-MB-231 cells. **(G)** Immunocytochemical analysis of GAGE protein expression in MDA-MB-231 and MCF-7 cells (*n* = 3) treated with the indicated concentrations of guadecitabine for 6 days. Mean GAGE staining intensity in the nuclei was quantified by immunofluorescence microscopy. (**H-I**) Western blot analysis of DNMT1-, DNMT3A- and DNMT3B-expression in MCF-7 (**H**) and MDA-MB-231 cell populations (I) treated with indicated concentrations of guadecitabine for 6 days. For each condition, two biological replicates are shown. Notably, DNMT3A and DNMT3B were undetectable in MDA-MB-231 cells and are therefore not shown. (**J**) Immunocytochemical analysis of DNMT1 protein expression in MDA-MB-231 and MCF-7 cells (*n* = 3) treated with the indicated concentrations of guadecitabine or vehicle for 6 days. Mean DNMT1 staining intensity in the nuclei was quantified by immunofluorescence microscopy. (**K**) Immunohistochemical analysis of Ki-67 expression (brown) in cells of guadecitabine- and vehicle-treated PDX-9228 and MDA-MB-231 tumors (*n* = 4). Five biological replicates were analyzed. Representative images are shown. Counterstain: Hematoxylin (blue). Size bars = 100 µM. (**L**) EdU labeling of MDA-MB-231 cells treated with 0.1 µM guadecitabine or vehicle for 6 days. The mean intensity of EdU in nuclei was quantified by immunofluorescence microscopy. The experiment was repeated twice with similar results. Statistical testing was performed using one-way ANOVA followed by Tukey’s multiple comparison testing. ns = non-significant. (**M**) RNA-sequencing analysis of MDA-MB-231 cells labeled with the PKH26 fluorophore and subsequently treated with 0.1 µM guadecitabine or vehicle for 5 days before cell sorting according to cell division-induced dilution of PKH26 levels in the cell membrane. P1 and P4 represents the cell populations with the fewest and highest duplications, respectively

Four breast cancer PDX models and the MDA-MB-231 xenograft model, all with infrequent baseline expression of CTAs (Figure S1) similar to patient tumors (Figure S2), were treated with the DNMTi guadecitabine (see Figure S3 for treatment schedules). Previously, these breast cancer models demonstrated potent activation of ERV expression and antiviral signaling in response to DNMTi treatment [17]. RNA-sequencing analysis of the PDX and MDA-MB-231 tumors showed that guadecitabine effectively induced a broad panel of CTA genes (Fig. 1A). To uncover potential intratumoral variations in response to the treatment, we stained the tumors for a panel of CTAs, specifically MAGE-A, GAGE, NY-ESO-1, and MAGE-C1. This approach revealed a high degree of intratumoral heterogeneity in DNMTi-induced CTA expression with only 1–25% positive cells (Fig. 1B-C). Comparable levels of heterogeneity were observed across both high (24.4 mg/kg; Figure S3) and low (2 mg/kg; Figure S3) dosage regimens of guadecitabine treatment (Fig. 1B and S4). Importantly, CTA-positive cells were scattered throughout the tumors (Figure S5), suggesting that the observed heterogeneity in response to DNMTi treatment was not attributable to differences in drug penetration or the microenvironment.

In vitro treatment of breast cancer cell lines (MDA-MB-231 and MCF-7) resulted in a similar heterogenous expression of CTAs, even at higher concentrations of guadecitabine (up to 3 µM, Fig. 1D-E) with minimal additional impact on CTA expression observed beyond 0.1 µM doses (Fig. 1F and G). To elucidate this variability, we examined DNMT depletion levels following guadecitabine treatment. Western blot analysis demonstrated effective depletion of the three catalytically active DNMT enzymes, DNMT1, DNMT3A, and DNMT3B, even at doses as low as 0.1 µM (Fig. 1H-I). Single cell analysis of DNMT levels using quantitative immunofluorescence confirmed that escalating the dose of guadecitabine beyond 0.1 µM had marginal additional impact on DNMTi levels (Fig. 1J). However, a more detailed inspection of DNMT levels in single cells unveiled a minor subset of cells retaining DNMT levels, even at high doses (up to 3 µM, Fig. 1J). The presence of this subset of seemingly DNMTi-resistant cells might partially account for the observed heterogeneity in CTA expression among treated cells. Nevertheless, the frequency of DNMT-depleted cells following guadecitabine treatment significantly surpassed the frequency of CTA-positive cells, implying the involvement of other mechanisms contributing to the intercellular variability in response to DNMTi.

Pyrimidine nucleoside analogs, such as guadecitabine, induce demethylation by depleting DNMT enzymes during DNA replication, making their activity dependent on cell division. Therefore, we investigated whether the intratumoral heterogeneity in DNMTi-induced CTA expression, observed in both tumors and in vitro cultures, could be attributed to differences in the rates of cell division among cancer cells. However, we found that the majority of cancer cells in DNMTi-treated PDX and MDA-MB-231 tumors were positive for the proliferation marker Ki-67 (Fig. 1K). Additionally, EdU nucleoside labeling suggested that all in vitro DNMTi-treated MDA-MB-231 cells underwent proliferation (Fig. 1L). Tracking the number of cell divisions in DNMTi-treated MDA-MB-231 cells revealed a correlation between the number of cell divisions and the level of CTA expression (Fig. 1M). Nevertheless, even cell populations undergoing a small number of divisions demonstrated a significant induction of CTA expression. Also, prolonged guadecitabine treatment of MDA-MB-231 cells (up to 28 days) did not increase CTA gene expression (Figure S6). These results indicate that the varied response to DNMTis among cells is not directly linked to their proliferative capacity.

### Single-cell sequencing reveals extensive transcriptional heterogeneity in response to DNMTi

**Fig. 2:**
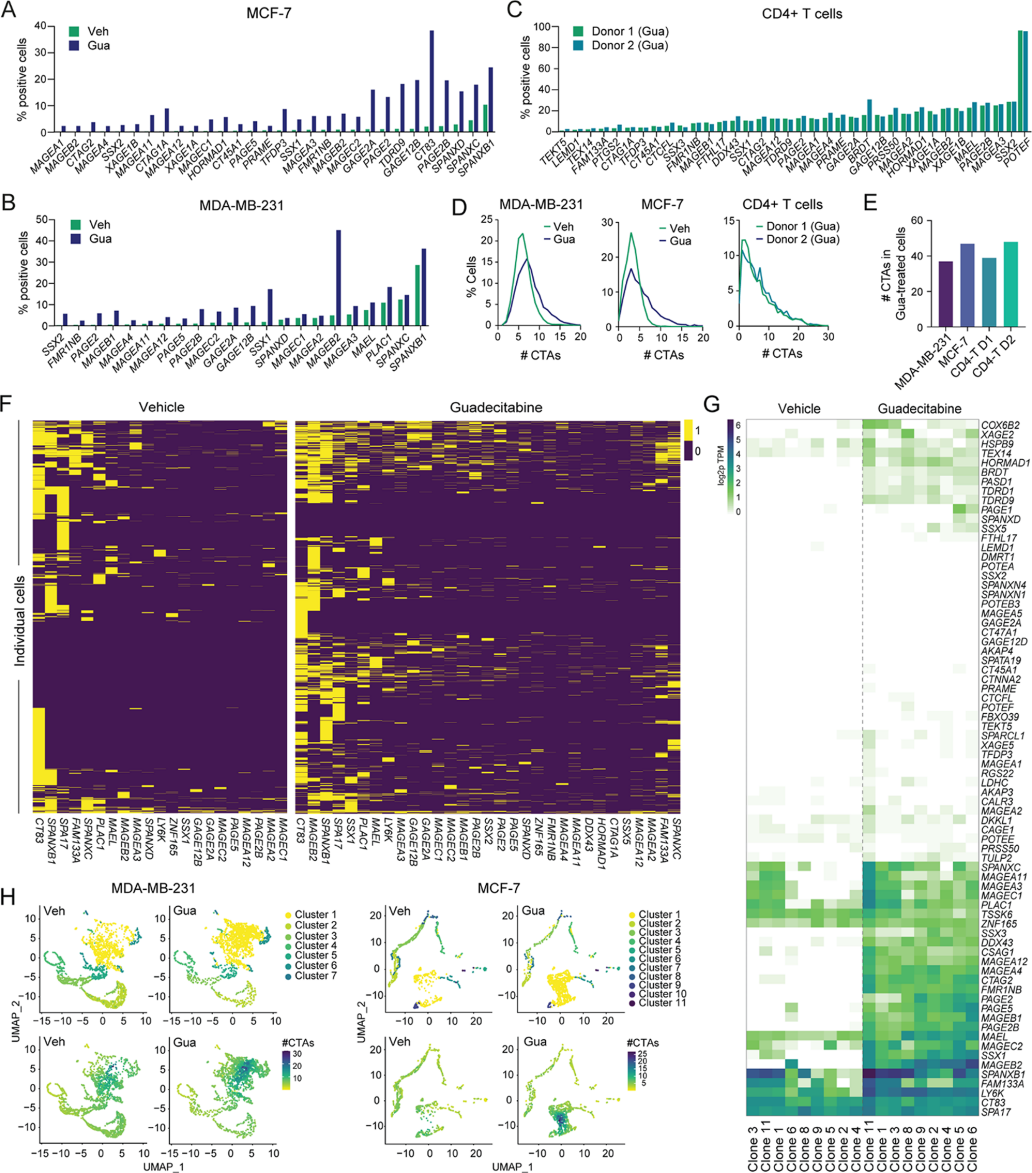
Single-cell sequencing demonstrates significant transcriptional heterogeneity in response to DNMTi treatment. (**A-E**) scRNAseq analysis of the expression of CTA genes in breast cancer cell lines treated with 0.1 µM guadecitabine (Gua) or vehicle (Veh) for four days, and in CD4 + T cells from two different donors, treated with guadecitabine according to the ALECSAT protocol [39]. Plots show percentages of cells expressing the different CTA genes (**A-C**; only Gua-responsive CTAs are shown), total number of expressed CTA genes in cell populations (**D**) and the number of CTA genes expressed per cell (**E**). (**F**) Heat map showing CTA gene expression in a panel of MDA-MB-231 single-cell clones treated with 0.1 µM guadecitabine or vehicle for four days. (**G**) Heatmap showing CTA expression profiles of individual guadecitabine- or vehicle-treated MDA-MB-231 cells (data as in A-E). Cells were clustered according to CTA expression. Yellow = expressed, Purple = not expressed (**H**) Clustering analysis of single-cell CTA gene expression in guadecitabine- and vehicle-treated MDA-MB-231 and MCF-7 cells (data as in A-E and G). UMAP dimensional reduction plots show clusters identified based on the combined analysis of guadecitabine and vehicle-treated cells (top), and feature plots show number of CTA genes expressed in cells from the different clusters (bottom)

To gain further insight into the varied responses of cancer cells to DNMTi treatment, we performed focused single-cell RNA sequencing (scRNAseq), investigating the expression of 83 CTA genes in MDA-MB-231 and MCF-7 cells treated with guadecitabine in vitro. This approach was designed to eliminate confounding factors from the tumor microenvironment, thus allowing a focused examination of cancer cell-intrinsic mechanisms. Consistent with our previous results, the analysis demonstrated a high diversity of CTA gene expression among DNMTi-treated cells, with expression of every CTA confined to a subset of cells (Fig. 2A-B). *MAGEB2* and *CT83* exhibited the most widespread expression upon treatment and was detected in 45% of MDA-MB-231 cells and 38% of MCF7 cells, respectively. However, on average, individual CTAs were expressed in only 10.6% and 9.3% of cells in DNMTi-treated MDA-MB-231 and MCF7 cultures, respectively (Fig. 2A-B). Moreover, individual DNMTi-treated cancer cells expressed only minor subsets of the total number of CTAs expressed across the cell population (Fig. 2D-E). On average, DNMTi treatment only increased the average number of CTA genes expressed in individual MDA-MB-231 and MCF7 cells from 6 to 8 and 3 to 7, respectively (Fig. 2E).

**Fig. 3:**
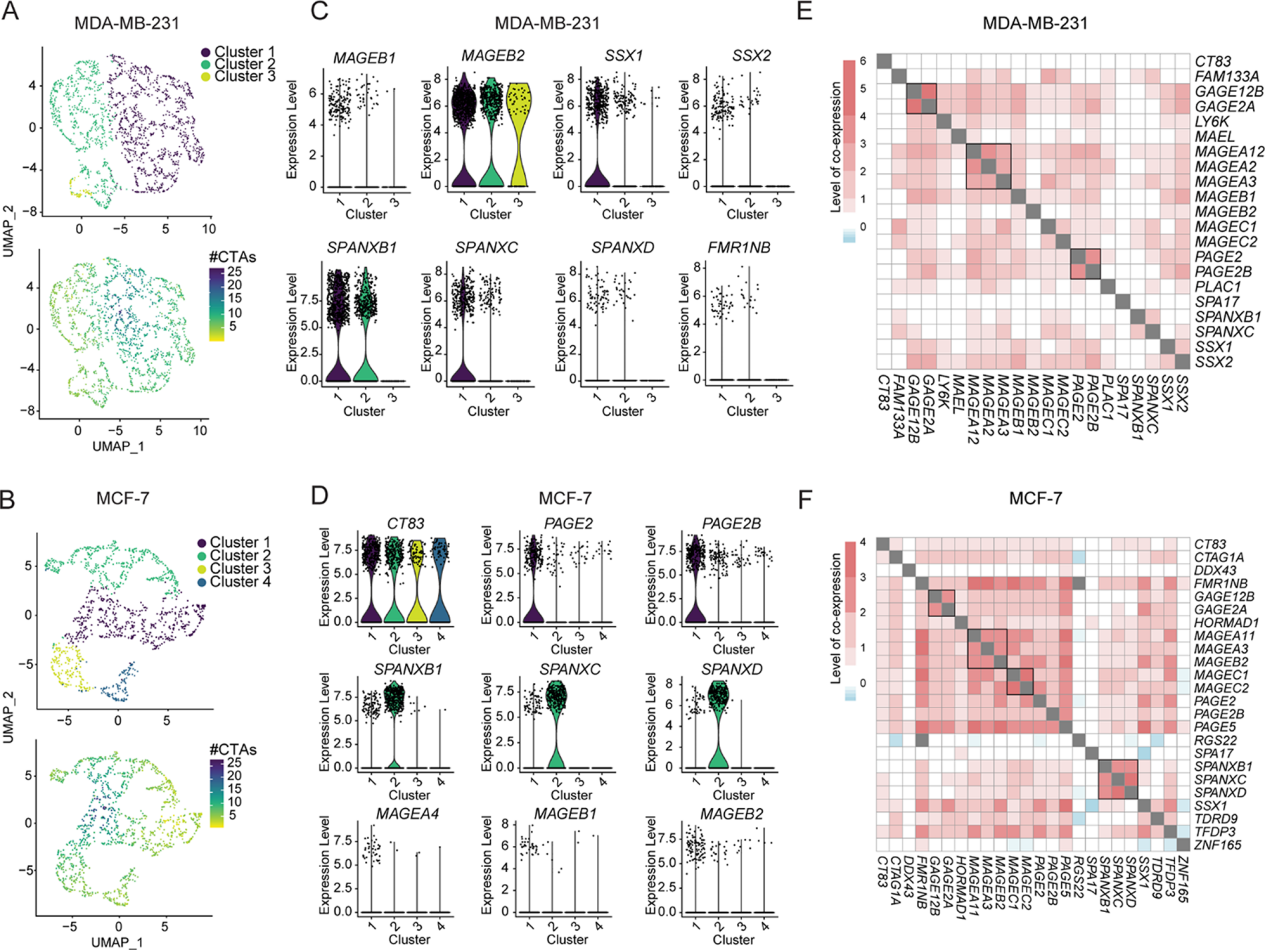
Lack of cell populations with distinct CTA gene expression profiles among DNMTi-treated cells. (**A-D**) scRNAseq analysis of the expression of CTA genes in DNMTi-responsive MDA-MB-231 (cluster 1; Fig. 2H) and MCF-7 (cluster 1; Fig. 2H) cells. (**A-B**) UMAP dimensional reduction plots show identified cell clusters (top) and number of CTA gene expression (bottom). (**C-D**) Expression of selected CTA genes in identified cell clusters. The expression of additional CTA genes in these clusters is shown in Figure S9. (**E-F**) Pairwise co-expression analysis of CTA genes in guadecitabine-treated MDA-MB-231 cells, based on scRNAseq data (as in Fig. 2A-E and G-F). Scales depict the level of co-expression (observed co-expression divided by expected co-expression based on the frequency of expression within tumors)

Substantial heterogeneity was observed in the subsets of CTAs expressed among individual DNMTi-treated cells in the MDA-MB-231 and MCF-7 populations. For instance, clustering analysis revealed distinct patterns in CTA expression; however, these patterns appeared to be predominantly shaped by variances in baseline CTA expression levels (e.g. *CT83*,* SPANXB1*,* SPA17*) rather than by the impact of DNMTi treatment on CTA expression (Fig. 2F). Instead, DNMTi appeared to induce the expression of highly distinct subsets of CTAs within individual cells, with no discernible patterns. Similarly, UMAP dimensional reduction analysis demonstrated distinct clusters of cells, mainly defined by differences in their baseline expression of CTAs (Fig. 2H and S7). For both MDA-MB-231 and MCF7 cells, most clusters displayed CTA expression profiles indicating a general unresponsiveness to DNMTi treatment. Responsive cells seemed to be predominantly found within a single cluster (cluster 1; Fig. 2H). Additional UMAP analysis of this cluster of responding cells could not demonstrate subsets of cancer cells with distinct DNMTi-induced CTA profiles (Fig. 3A-B). Collectively, the heat map and dimensional reduction analysis suggested a high level of heterogeneity in the response to DNMTi among cancer cells.

A comparable heterogeneous pattern in the activation of CTA genes was also noted in non-cancerous cells in the form of primary CD4 + T cells following treatment with DNMTi. Similar to the cancer cells, single-cell sequencing showed that individual CTA genes were activated only in a small subset of CD4 + T cells (Fig. 2C-D) and individual CD4 + T cells expressed seemingly random subsets of the CTA genes in the DNMTi-treated cell population (Figure S8). The heterogeneous response to DNMTi of this genetically homogenous population of T cells, demonstrated that the observed variability in CTA gene activation among cells (primary T cells and cancer cells) was independent of genetic variation. This was supported by RNA-seq analysis of DNMTi-treated MDA-MB-231 clonal cultures, showing a consistent DNMTi response among different cancer cell subsets (Fig. 2G).

Despite this high variability in the response to DNMTi, there was a tendency for co-expression of genes belonging to distinct CTA families, including MAGE, PAGE, SPANX and SSX, in both MDA-MB-231 and MCF-7 cells (Fig. 3C-D and S9). To thoroughly explore potential transcriptional associations among CTAs, we conducted pairwise comparisons of their expression patterns among DNMTi-treated MDA-MB-231 and MCF-7 cells (Fig. 3E-F). While most CTAs showed little correlation in their expression, we confirmed a notable trend toward co-expression among evolutionarily and structurally related genes. This pattern indicates that such genes are regulated by common enhancers, maintained within identical regulatory domains, or are controlled by common regulatory factors.

### DNMTi treatment leads to heterogeneous and random DNA demethylation

**Fig. 4:**
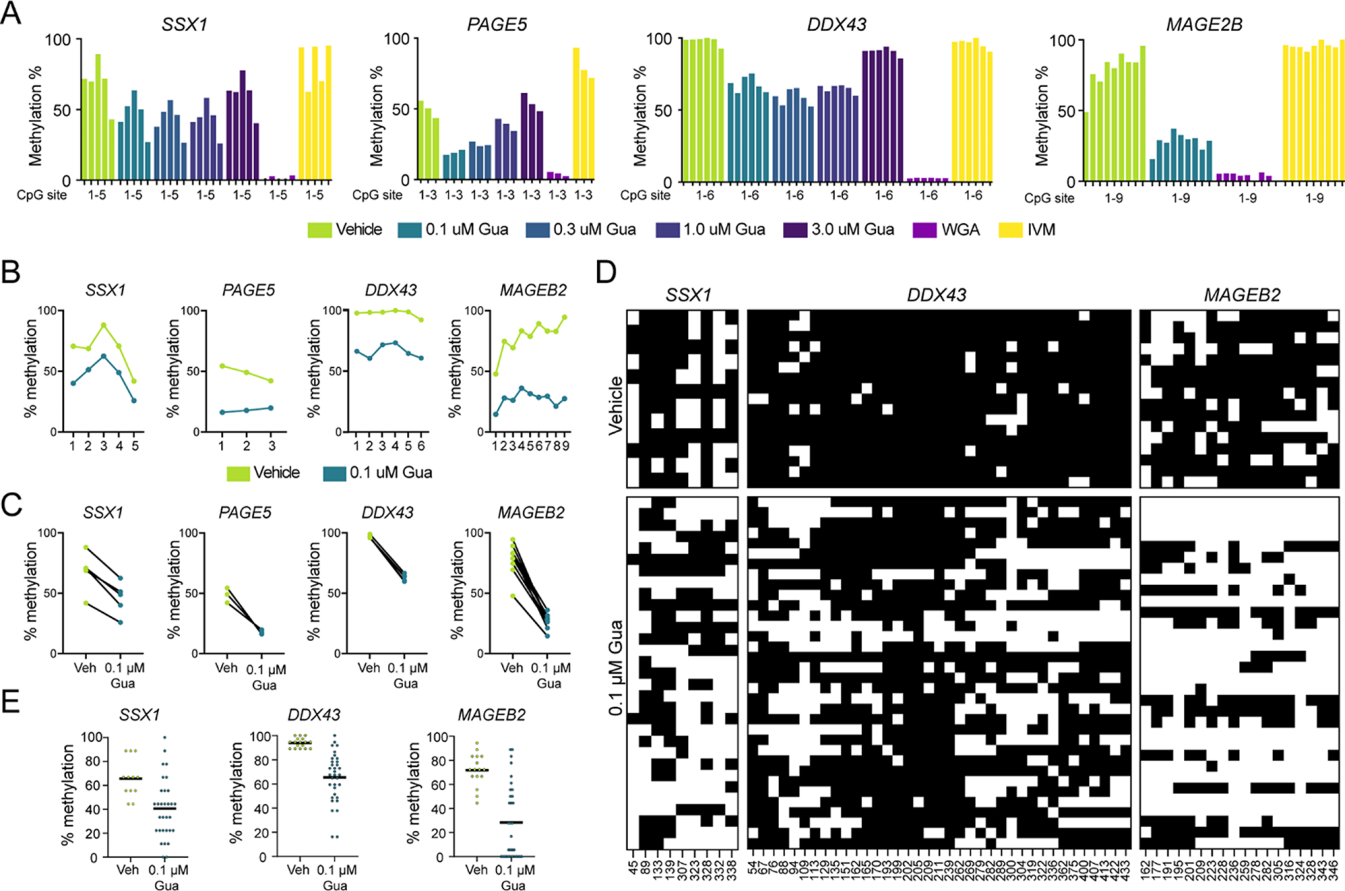
Stochastic and incomplete demethylation by DNMTi treatment. (**A-C**) Pyrosequencing analysis of methylation levels of different CpG sites located proximal to the transcription start site of selected CTA genes of MDA-MB-231 cells. Cells were treated with indicated concentrations of guadecitabine (Gua) or vehicle (Veh) for four days. WGA = whole genome amplification (no CpG methylation expected). IVM = in vitro methylated genomic DNA (complete CpG methylation expected). Plots show CpG site methylation levels (average of two biological replicates). (**D-E**) Analysis of methylation levels of individual CTA promoters of selected CTA genes. Promoter sequences were PCR-amplified from bisulfite-converted gDNA, obtained from MDA-MB-231 cells treated with 0.1 µM guadecitabine or vehicle for four days, and individual promoters were cloned and sequenced. Plots show the methylation status of CpG sites (**D**; black = methylated, white = non-methylated) and the average methylation level for each individual promoter sequence (**E**)

To elucidate the mechanistic basis of the heterogeneous response to DNMTi treatment at the single-cell level, we conducted an in-depth analysis of the methylation levels of CpG sites within the promoters of a panel of CTA genes, which showed heterogenous expression in DNMTi-treated MDA-MB-231 cells (Fig. 4). Examination of bulk samples revealed a remarkably uniform reduction in gene methylation, averaging a 30–40% decrease across CpG sites and promoters following DNMTi treatment (Fig. 4A-C). This consistency suggested that the impact of DNMTi treatment was broadly similar across CpG sites. Furthermore, the level of demethylation remained reasonably stable over a broad range of guadecitabine concentrations (0.1-3.0 µM; Fig. 4A), suggesting optimal efficacy at a relatively low concentration (0.1 µM). This was consistent with the depletion of DNMTs and induction of CTA expression, reaching a plateau at similar concentrations (Fig. 1E-J). High concentrations of guadecitabine (3.0 µM) tended to induce lower levels of demethylation (Fig. 4A), possibly due to increased cytotoxicity. A highly similar demethylation pattern in response to DNMTi treatment was observed for a LINE-1 element, suggesting that these results reflect a genome-wide phenomenon (Figure S10).

To further investigate promoter methylation at the single-cell level, we cloned and sequenced promoters of individual CTA genes from DNMTi-treated MDA-MB-231 cells. This analysis revealed a highly heterogeneous pattern, demonstrating the variable effects of DNMTi treatment on CpG sites across promoters in distinct cells (Fig. 4D), which suggests a stochastic process of demethylation at sites critical for gene repression. This variability closely reflects the heterogeneous expression patterns of CTA genes induced by DNMTi treatment observed in breast cancer tumors and cell lines (Figs. 1, 2 and 3). Furthermore, this analysis showed a range of demethylation responses among promoters in individual cells, with some cells displaying pronounced demethylation and others exhibiting low or no demethylation (Fig. 4E). This variability points to differential cellular susceptibilities to DNMTi, possibly due to differences in metabolism or drug uptake.

Interestingly, our analysis showed that the extent of demethylation in CTA promoters in DNMTi-treated MDA-MB-231 cells (Fig. 4A-C) greatly exceeded the corresponding level of CTA gene activation (Fig. 2B). For example, after treating cells with 0.1 µM guadecitabine, demethylation at CpG sites within the *PAGE5* promoter was 80–84%, yet *PAGE5* expression was detected in only 6% of the cells. Similarly, the *SSX1* promoter exhibited a methylation loss of 38–75%, while the gene was expressed in just 17% of DNMTi-treated cells. This notable discrepancy suggests that the activation of gene expression may require demethylation at multiple CpG sites concurrently, and/or it may indicate the presence of other inhibitory epigenetic mechanisms, such as repressive histone modifications.

### HDAC inhibition reduces expression heterogeneity in response to DNMTi treatment

**Fig. 5:**
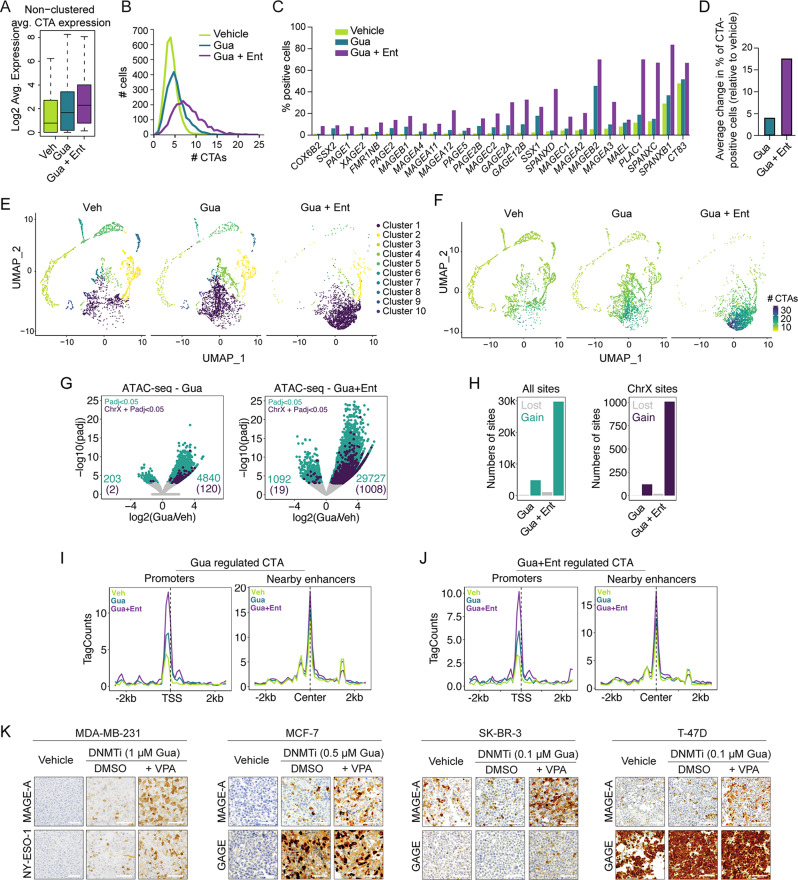
HDACi reduces heterogeneity in response to DNMTi. (**A-F**) scRNAseq analysis of CTA gene expression in MDA-MB-231 cells treated with either 0.1 µM guadecitabine (Gua) for four days, 0.1 µM guadecitabine for four days followed by treatment with 1µM entinostat (Ent) for two days, or vehicle (Veh). Plots show the average level of CTA expression (**A**), number of CTA genes expressed per cell (**B**), frequency of CTA-positive cells (**C**) and the average change in frequency of cells positive for individual CTAs (**D**). (**E-F**) Clustering analysis of cells based on single-cell CTA gene expression (data as in A-D). UMAP dimensional reduction plots show identified clusters (E), and feature plots show the number of CTA genes expressed in cells from the different clusters (**F**). (**G-J**) ATAC-seq analysis of the chromatin structure of MDA-MB-231 cells treated as in A-F. DiffBind was used to identify significantly different accessible regions (Padj < 0.05) (**G** and **H**). Changes in accessibility at promoters and nearby enhancers (+/-50 kb) in response to treatment are shown as average line plots (**I** and **J**). (**K**) Immunohistochemical staining of GAGE and MAGE-A CTAs (brown) in various breast cancer cell lines (MDA-MB-231, MCF-7, SK-BR-3) following treatment with 0.1 µM guadecitabine for four days, 0.1 µM guadecitabine for four days followed by treatment with 1 mM valproic acid (VPA) for two days, or vehicle. Counterstain: Hematoxylin (blue). Size bars = 100 µM. Representative images are shown

DNA methylation and histone deacetylation are fundamental mediators of transcriptional repression, acting independently yet interconnectedly [48]. While histone deacetylase inhibitors (HDACis) have limited efficacy as single agents in activating methylation-dependent genes, such as CTAs, they have been shown to enhance the effects of DNMTis on the activation of these genes (Figure S11) [49–51]. Therefore, scRNAseq was employed to assess the efficacy of HDACis in reducing the heterogeneity in CTA expression observed in breast cancer tumors and cell lines following DNMTi treatment. Supplementing DNMTi with HDACi increased the overall induction of CTA expression in MDA-MB-231 cells (Fig. 5A) and markedly increased the number of CTAs expressed per cell (Fig. 5B) as well as the fraction of cells expressing individual CTAs (Fig. 5C). For example, the proportion of cells expressing *SPANXB1* and *MAGEB2* rose from 36 to 83% and 45 to 69%, respectively, with the addition of HDACi to DNMTi. Overall, the combined treatment increased the average frequency of CTA-positive cells by 17.3-fold compared to the vehicle control, whereas DNMTi alone resulted in only a 4-fold increase (Fig. 5D). This ability of HDACi to reduce the variability in DNMTi-induced CTA expression was further highlighted by a more uniform CTA expression pattern across cells (Fig. 5E), attributed to the increased likelihood of cells sharing similar CTA profiles as the number of expressed CTA genes increased (Fig. 5F). As expected, HDACi single treatment did not reduce promoter methylation levels of CTA genes (Figure S12), suggesting that the additive effect of HDACi to DNMTi was due to targeting of DNA methylation-independent suppressive functions. Furthermore, the frequency of cells expressing a specific CTA (Fig. 5C) did not exceed the level of demethylation (Fig. 4A) in the population of DNMTi + HDACi treated cells, suggesting that HDACi potentiates the transcriptional activation of genes primed by DNMTi.

To determine whether the enhanced induction of CTA expression by DNMTi combined with HDACi, compared to DNMTi alone, was due to a stronger effect on chromatin accessibility, we performed ATAC-seq (Assay for Transposase-Accessible Chromatin using sequencing) analysis. The results showed that the DNMTi and HDACi combination was significantly more potent than DNMTi alone in opening the chromatin structure of MDA-MB-231 cells, both genome-wide (Fig. 5G-H) and with respect to CTA gene promoters (Fig. 5I-J). This suggests that HDACis are essential partners to DNMTis in inducing homogenous expression of CTA genes in cancer cell populations by increasing chromatin accessibility.

Consistent with these findings, the addition of HDACi to DNMTi significantly enhanced CTA expression at the protein level across various breast cancer models (Fig. 5K), further highlighting the potential of the combination treatment to elicit more robust and widespread drug responses in tumors.

## Discussion

DNMTis have attracted substantial attention for their potential to alter key aspects of tumor biology. Despite encouraging preclinical results, the translation of DNMTis into the clinical management of solid cancers has produced only modest outcomes. To elucidate resistance mechanisms and advance the clinical progress of DNMTis in solid tumor management, we studied the intratumoral heterogeneity of breast cancers in their response to these agents, using CTA genes as a proxy for methylation-dependent gene silencing. Despite the robust induction of CTA gene expression in breast cancer PDX tumors and cell lines upon DNMTi treatment, our investigation revealed a marked heterogeneity at the single-cell level, even with effective DNMT enzyme depletion.

Our findings carry important implications for the clinical implementation of DNMTis. The ability of DNMTis to induce CTA expression offers promising targets for immunotherapeutic strategies, including cancer vaccines or T-cell therapies. Yet, the heterogeneity in CTA activation within tumors might allow for the survival of antigen-negative subclones, potentially undermining anti-tumor immunity and leading to treatment resistance. Another critical outcome of DNMTi therapy is the reactivation of tumor suppressor genes, leading to cell cycle arrest and apoptosis in cancer cells. However, the sustained advantage of this effect might be significantly challenged by the evident heterogeneity in the activation of genes induced by DNMTi treatment. This variability may likewise affect other promising clinical applications of DNMTis, such as their potential to support endocrine therapy or other targeted approaches, thus limiting their broader application in clinical settings.

Our cell line studies identified diverse mechanisms behind the observed heterogeneity in the response to DNMTi. First, even with concentrations of guadecitabine up to 3 µM, only partial demethylation of CTA promoter CpGs was achieved, averaging 40% across multiple sites (Fig. 4A-C). Detailed sequencing of these promoters revealed a stochastic pattern of demethylation, potentially leading to inconsistent activation of CTA genes across individual cells. Second, we observed differential overall demethylation levels among cells (Fig. 4D-E), suggesting variable DNMTi sensitivity, which could stem from intercellular differences in mechanisms that affect intracellular drug metabolism and availability [52–55]. Remarkably, the consistent response to DNMTi among clonally derived MDA-MB-231 cultures, along with the observed heterogeneity among DNMTi-treated primary CD4 + T cells, implies that susceptibility to DNMTi treatment is not solely dependent on genetic aberrations. Third, we observed that DNMTi-mediated promoter demethylation often exceeded actual CTA gene expression, pointing to additional epigenetic barriers to gene activation. Our findings further confirm the interplay between DNA methylation and histone deacetylation in transcriptional repression, showing that combining HDACi with DNMTi enhances chromatin accessibility of CTA promoters, enhances the proportion of CTA-positive cells and substantially mitigates CTA expression variability, suggesting that HDAC-mediated repression plays a significant role in the heterogeneous DNMTi response.

In conclusion, our study highlights extensive heterogeneity in the response to DNMTi treatment among both solid tumors and cell lines, providing insight into the challenges of DNMTi monotherapy and the transient nature of responses in many patients. Although the study was carried out using breast cancer models, the stochastic demethylation and the interplay between DNA methylation and histone acetylation, which we identified as key mechanisms driving the heterogeneous response to DNMTi treatment, are likely conserved across various cancer types, regardless of stage, clinical characteristics, genetic and epigenetic differences. Our findings advocate for the synergistic use of HDACi and DNMTi as a strategy to improve therapeutic outcomes in solid cancer treatment, thereby suggesting a potent avenue to overcome resistance and enhance efficacy. The combination of HDACi and DNMTi has demonstrated promising anti-tumor effects in preclinical models [22, 56, 57]; however, its clinical potential remains to be fully established.

## Electronic supplementary material

Below is the link to the electronic supplementary material.

Supplementary Material 1

Supplementary Material 2

Supplementary Material 3

## Data Availability

No datasets were generated or analysed during the current study.

## Abbreviations

CTAs: Cancer/testis antigens
DNMTs: DNA methyltransferases
HDACis: Histone deacetylase inhibitors
ERVs: Human endogenous retroviruses
scRNAseq: Single-cell RNA sequencing
