# Supplementary material for: Stochastic demethylation and redundant epigenetic suppressive mechanisms generate highly heterogeneous responses to pharmacological DNA methyltransferase inhibition
Source: J Exp Clin Cancer Res. 2025 Jan 23;44:21. doi: 10.1186/s13046-025-03294-x (PMC11755921; doi:10.1186/s13046-025-03294-x)
Supplement: Supplementary file 1 — Supplementary Material 1 [file 13046_2025_3294_MOESM1_ESM.docx]

**Pyrosequencing primers**

| **Target gene** | **Sequence (5’-3’)** |
| --- | --- |
| SSX1 forward primer | GGGATTTTTTTTTAGGAATTTTGTAGTG |
| SSX1 reverse primer | ATTCAAATAATCCTCCAACTATAACCT |
| SSX1 sequencing primer | TTTTTTTTAGGAATTTTGTAGTGA |
| PAGE5 forward primer | GAAGAGTTTGTGGTTTAGGTTTTTTATAG |
| PAGE5 reverse primer | ACCTCTCCTTACAAAACTCTAC |
| PAGE5 sequencing primer | GTTTTTTATAGAGGTAGGAAATT |
| DDX43 forward primer | GGTTAGGTGGTGTAGAGTT |
| DDX43 reverse primer | ACCCACCTAAAAACCTTAAAAACTCCT |
| DDX43 sequencing primer | GGTGGTGTAGAGTTG |
| MAGE2B forward primer | TAGATATTGAGGTGAGGATTTTTAGT |
| MAGE2B reverse primer | TTTCAAAACCAAAATCAAAATAACTCACAT |
| MAGE2B sequencing primer | TGAGGATTTTTAGTGGAGA |

**Sanger sequencing primers**

| **Target gene** | **Sequence (5’-3’)** |
| --- | --- |
| SSX1 forward | GGGATAGAATTTTTTTAAAATGGAG |
| SSX1 reverse | AACTAAAAATCCCTAAAACCCCACT |
| MAGEB2 forward | GGTTTGTGAGGTTTAGGTAGGGGTG |
| MAGEB2 reverse | ACCTCTCAAAACCAAATACAAAAAC |
| DDX43 forward | GATTGGTTAAAATTGGGAAAGTTT |
| DDX43 reverse | CTAACCCCACCTATCCTACCCTAC |

**Antibodies**

| **Reactivity** | **Clone** | **Company** | **Application*** |
| --- | --- | --- | --- |
| pan-GAGE | M3 | Produced in house [39] | IHC 1:100  IF 1:100 |
| pan-MAGE-A | 6C1 | Santa Cruz Biotech | IHC 1:500 |
| MAGE-C1 | CT7-33 | Santa Cruz Biotech | IHC 1:500  IF 1:100 |
| NY-ESO-1 | E978 | Santa Cruz Biotech | IHC 1:100 |
| DNMT1 | ab188454 | Abcam | IF 1:500 |

* IHC = immunohistochemistry; IF = Immunofluorescence
