# Supplementary material for: Stochastic demethylation and redundant epigenetic suppressive mechanisms generate highly heterogeneous responses to pharmacological DNA methyltransferase inhibition
Source: J Exp Clin Cancer Res. 2025 Jan 23;44:21. doi: 10.1186/s13046-025-03294-x (PMC11755921; doi:10.1186/s13046-025-03294-x)
Supplement: Supplementary file 3 — Supplementary Material 3 [file 13046_2025_3294_MOESM3_ESM.docx]

**Figure S1. Expression of selected CTAs in breast cancer patient-derived xenograft models.** RNA-sequencing analysis of CTA expression in breast cancer PDX tumors. Data shown as log_2_(TPM+1).

**Figure S2. Expression of selected CTAs in breast cancer tumors.** Gene expression levels for triple-negative breast cancer samples obtained from the RNAseq (polyA+ IlluminaHiSeq) platform of The Cancer Genome Atlas (TCGA) breast invasive carcinoma (BRCA) gene expression dataset (n=133). Data shown as log_2_(RSEM+1).

**Figure S3. Schedule for treatment of xenograft mice with guadecitabine.** Mice were grafted with TNBC PDX models or the MDA-MB-231 cell lines and treated as indicated.

**Figure S4. Immunohistochemical statining of selected CTAs in guadecitabine or vehicle treated mice grafted with MDA-MB-231 cells using the low-dose treatment schedule (see Figure S3).**

MDA-MB-231 (low dose)


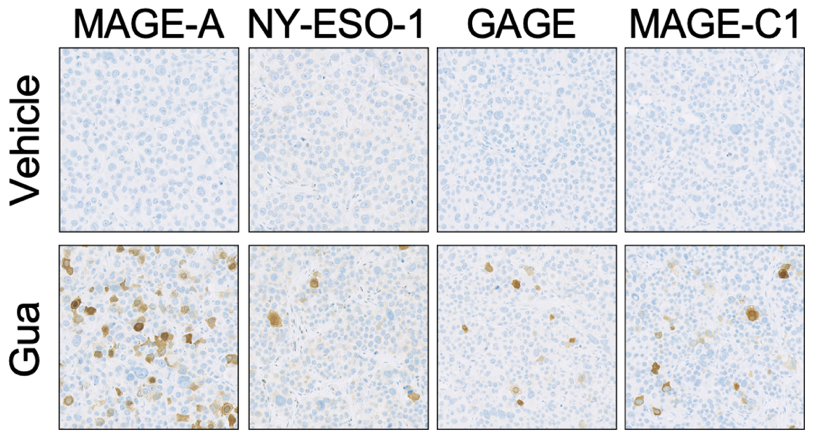

**Figure S5. Pictures of tumors from guadecitabine-treated mice.**

Representative picture of GAGE staining in a PDX-9228 tumor treated with guadecitabine using the low dose schedule.


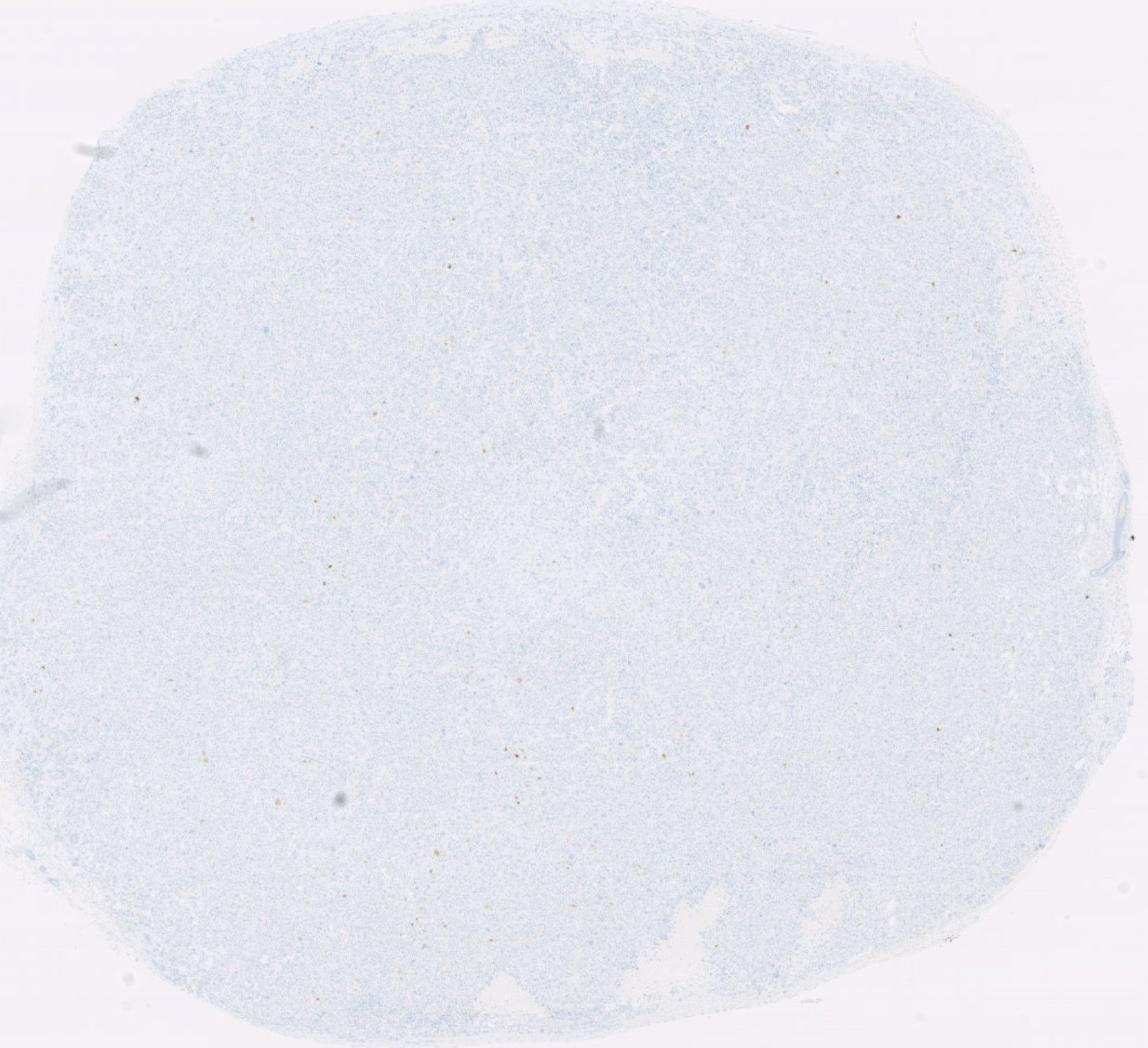


Representative picture of MAGE-A staining in a PDX-9228 tumor treated with guadecitabine using the low dose schedule.


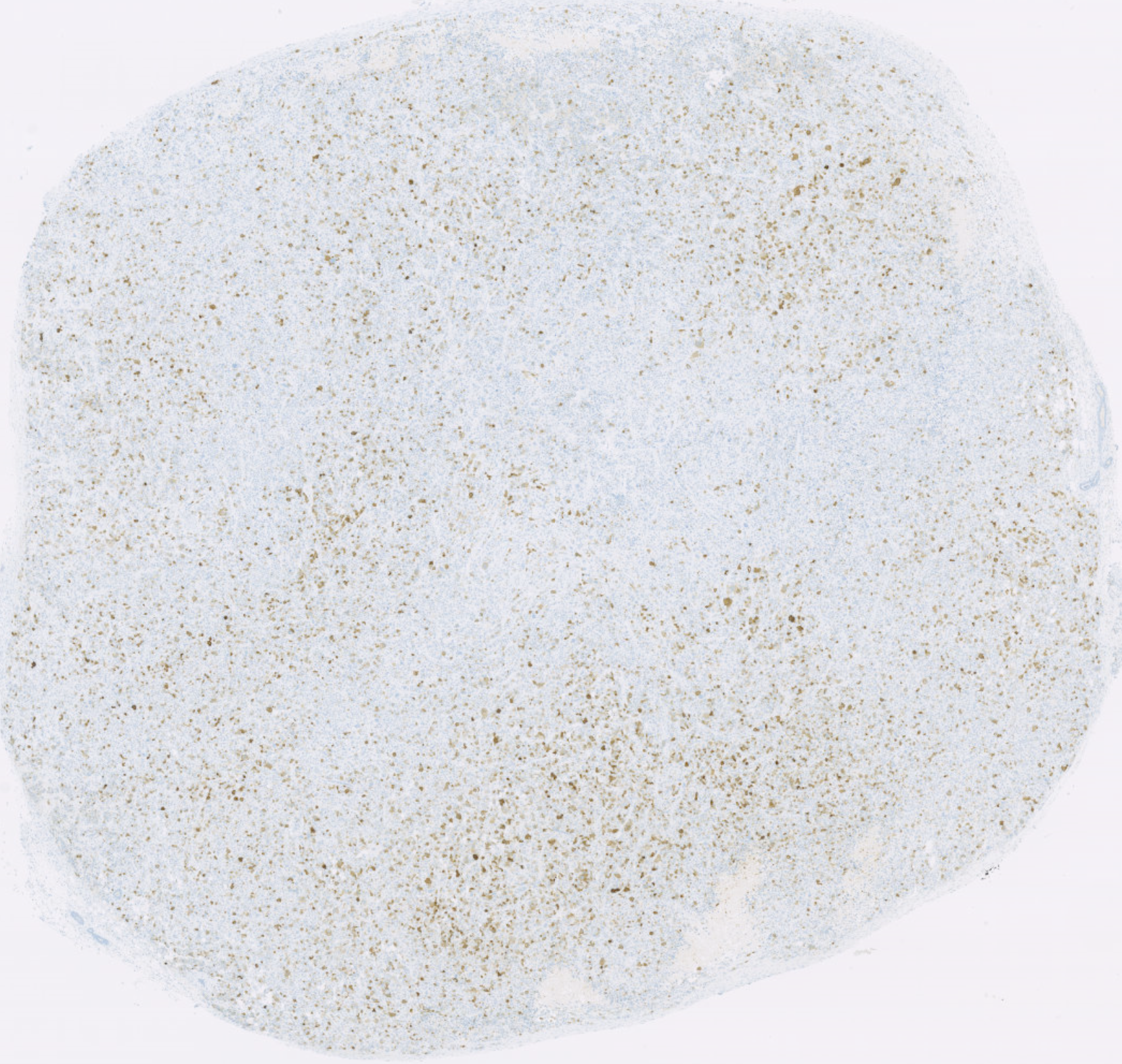


Representative picture of NY-ESO-1 staining in a PDX-9228 tumor treated with guadecitabine using the low dose schedule.


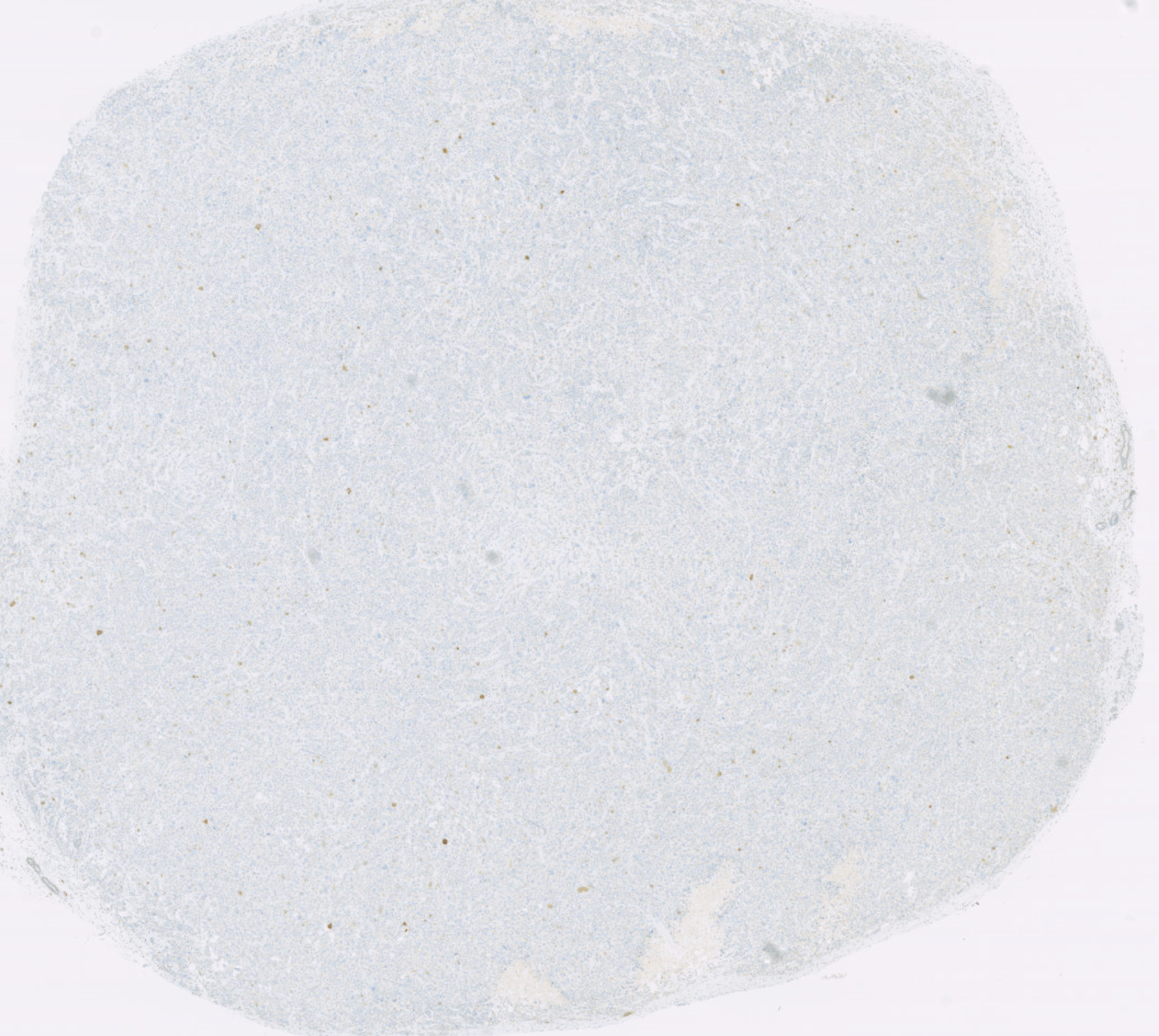


Representative picture of MAGE-C1 staining in a PDX-9228 tumor treated with guadecitabine using the low dose schedule.


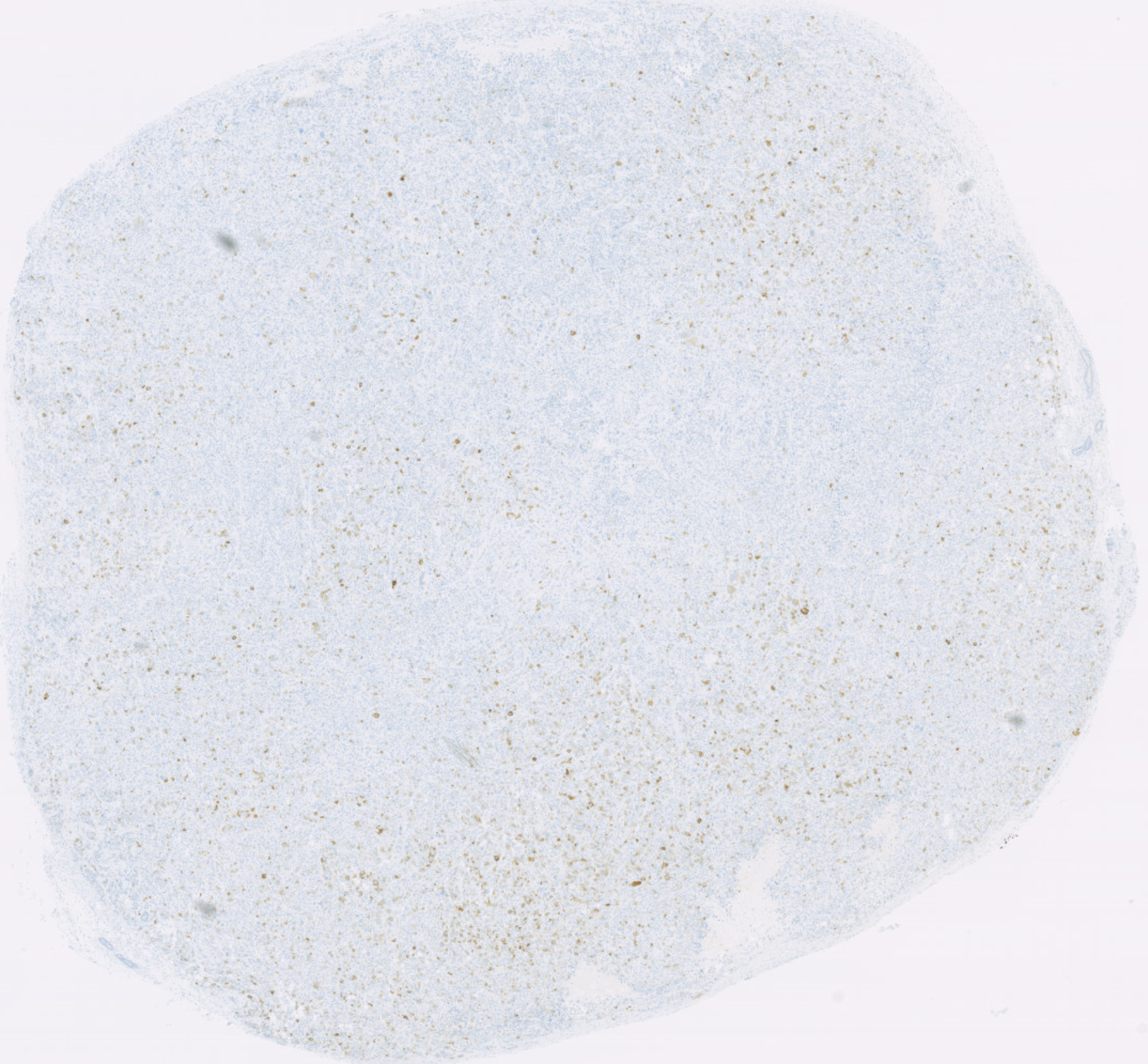


**Figure S6. The effect of long-term treatment on CTA expression.** MDA-MB-231 cells were continuously treated with 0.1 µM guadecitabine or vehicle (n=3). No statistically significant variances were observed in the level of MAGEB2 expression among guadecitabine-treated cells on the respective days, as determined by one-way ANOVA followed by Tukey's multiple comparison test.

**Figure S7. UMAP dimensional reduction analysis.** Clustering analysis of single-cell CTA gene expression in guadecitabine- and vehicle treated MDA-MB-231 and MCF-7 cells. UMAP dimensional reduction plots show clusters identified based on the combined analysis of guadecitabine and vehicle-treated cells and feature plots show the expression of selected CTA genes.

**Figure S8. CTA expression profiles of individual guadecitabine-treated CD4+ cells.** Heatmaps show CTA expression profiles of individual guadecitabine-treated CD4+ cells from two different donors. Cells were clustered according to CTA expression. Yellow = expressed, Purple = not expressed

Donor 1

**
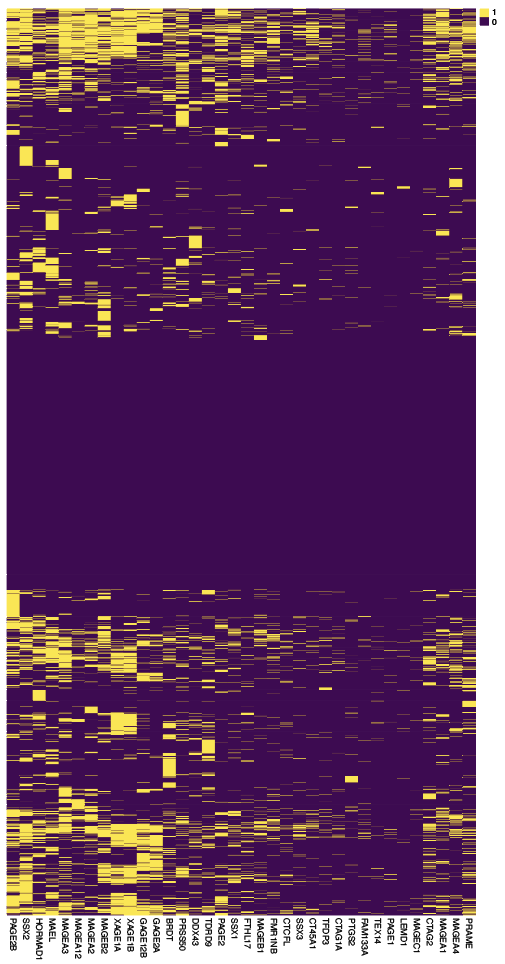
**

Donor 2


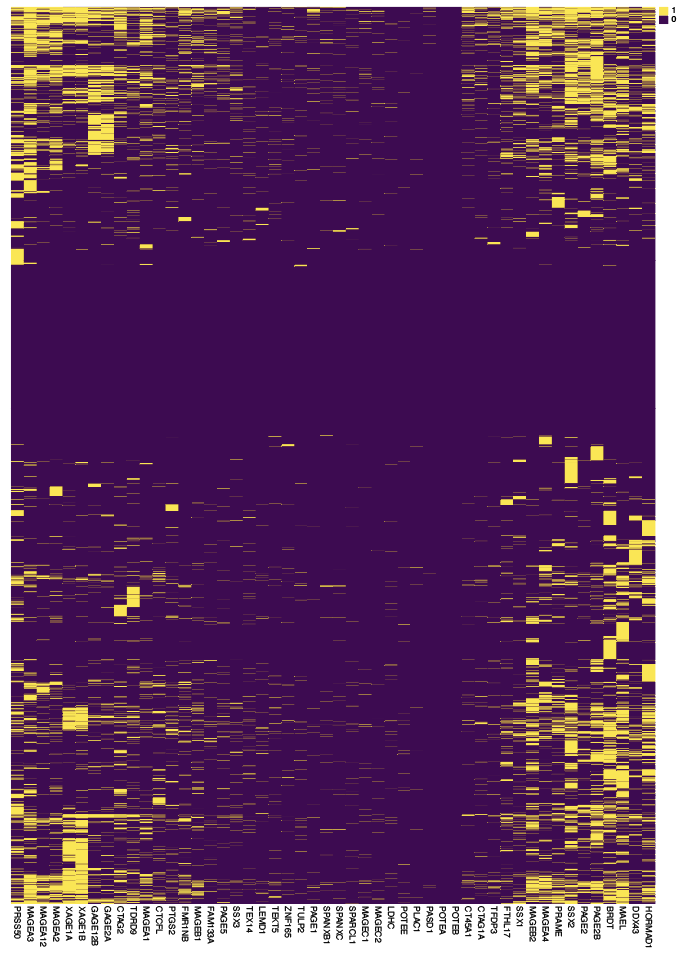


**Figure S9. Expression levels of additional CTA genes in clusters identified from UMAP dimensional reduction plots (Figure 3A-B) of guadecitabine-responding MDA-MB-231 and MCF-7 cells.**

**Figure S10.** **The effect of DNMTi on LINE-1 methylation.** Pyrosequencing analysis of methylation levels of different CpG sites located in a LINE-1 sequence of MDA-MB-231 cells. Cells were treated with indicated concentrations of guadecitabine (Gua) or vehicle (Veh) for four days. WGA = whole genome amplification (no CpG methylation expected). IVM = in vitro methylated genomic DNA (complete CpG methylation expected). Plots show CpG site methylation levels (average of two biological replicates).

**
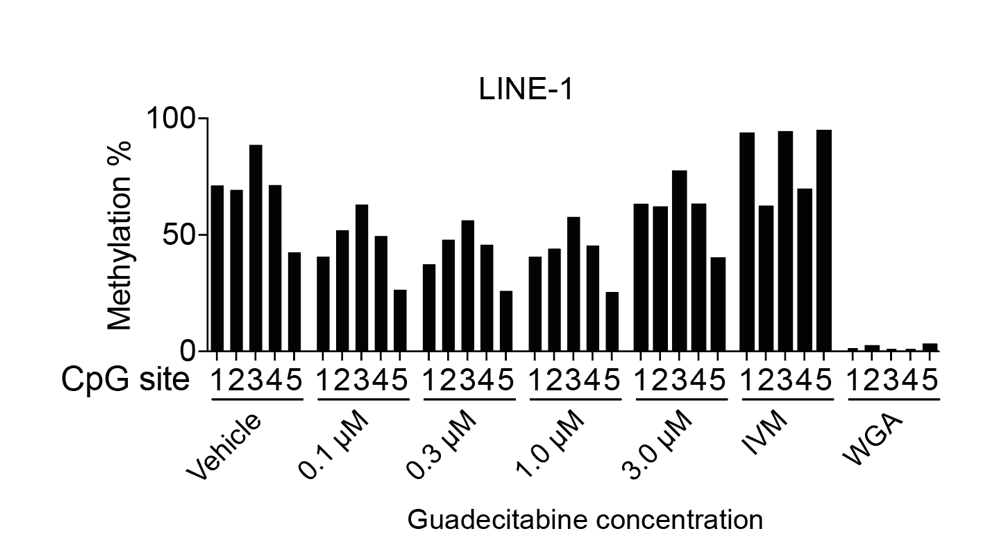
**

**Figure S11. The effect of combined DNMTi- and HDACi-treatment on expression of selected cancer/testis antigen genes.** Statistical test: One-way ANOVA followed by Tukey’s multiple comparison test. Significant levels relative to untreated. ns = non-significant. ns = non-significant. **** P < 0.00001; *** P < 0.0001; ** P < 0.001; * P < 0.01.

**Figure S12. Pyrosequencing analysis of CpG sites in promoters of selected cancer/testis antigen genes from cells treated with DNMTi or DNMTi+HDACi.** Data represent the average of two replicates.
